# Real-Time Laser Focus Tracking for Compressive Testing: Modular Rust Architecture with OPC UA and Web GUI for Laser Ultrasonics

**DOI:** 10.3390/s26185927

**Published:** 2026-09-19

**Authors:** Konstantin Manuel Prabitz, Marco Schwarz, Bernhard Reitinger, Florian Rudinger, Alexander Walzl, Martin Stockinger

**Affiliations:** 1 Chair of Metal Forming, Technical University of Leoben, Franz Josef-Straße 18, 8700 Leoben, Austria; 2 Research Center for Non Destructive Testing GmbH, Altenbergerstrasse 69, 4040 Linz, Austria

**Keywords:** laser-ultrasonics, Gleeble 3800, compression testing, laser-focus tracking, real-time positioning, analogue-to-digital conversion, Rust, OPC UA, thermo-mechanical testing

## Abstract

**Highlights:**

**What are the main findings?**

- Demonstration of an integrated Gleeble-laser-ultrasonics platform providing non-contact, in situ elastic/defect measurements during hot compression testing.
- A Rust-based control system with a custom analogue-to-digital converter reduced control-loop latency from ~130 ms to ~27 ms, improving laser-focus tracking on deforming specimens.

**What are the implications of the main findings?**

- Providing a reliable coupling of stress–strain data with ultrasonic signals leads to quantitative, real-time characterisation under high-temperature deformation.
- A modular architecture (OPC UA, parallel analogue-to-digital converters, linear stages) provides a scalable template leading to retrofit legacy thermo-mechanical simulator with advanced sensing and control.

**Abstract:**

Compression testing combined with laser ultrasonics enables non-destructive, in situ material characterisation during thermo-mechanical loading, but the shortening and radial expansion of the specimen displace the laser focal points during deformation and therefore require active repositioning of the laser-ultrasonic optics. This work addresses this engineering problem by providing a modular positioning-control system that couples a Gleeble 3800 thermo-mechanical simulator and a laser-ultrasonic measurement unit. The Gleeble provides two analogue shaft-position signals, which are digitised and used as real-time reference signals for stage-position control. The excitation and detection optics are mounted on motorised Zaber linear stages; two stages translate the optics along the compression direction, while a third perpendicular stage adjusts the detection focus to compensate radial specimen expansion and the limited depth of field of the detection optics. The control software was implemented in Rust and computes the stage target positions from the digitised Gleeble voltages output using user-configurable mathematical expressions. It further provides process-data access through an embedded OPC UA server and a browser-based graphical user interface through an integrated HTTP server. A custom ADC-based acquisition chain was introduced because the built-in ADC inputs of the Zaber controllers showed a significant noise level when used for the Gleeble shaft-position signals. Profiling showed that the control-loop runtime was dominated by ADC read/write operations; by reorganising acquisition into a parallel setup, the mean control-loop time was reduced from approximately 130 ms to approximately 27 ms. The resulting system provides an active, configurable, and retrofit-capable link between Gleeble compression testing and laser-ultrasonic measurement. It improves the temporal response available for laser-focus tracking on deforming specimens and provides a foundation for future quantitative validation of tracking error, positioning uncertainty, and LUS signal quality during real compression experiments.

## 1. Introduction

In metal forming, compression testing is a fundamental method to evaluate material characteristics under uniaxial compressive loading. It is essential for materials that primarily experience compressive stresses in service, such as forming tools or bulk materials. During a test, a specimen is mounted between rigid dies, and a controlled force or displacement is applied. Key quantities such as displacement, force response, and temperature are recorded, often under various thermal conditions. The resulting stress–strain data is used to determine properties including compressive strength, elastic modulus, yield behaviour, and strain hardening or softening. It can also provide insight into time-dependent phenomena like creep and microstructural evolution during plastic deformation.

A primary challenge in compression testing is maintaining homogeneous uniaxial deformation, as friction between the dies and the specimen can cause non-uniform “barrelling” [1]. This effect can be mitigated through careful friction control, optimised specimen geometry and well-established testing practices.

Specimens are typically cylindrical with a height-to-diameter ratio of less than 1.5 (H/D < 1.5) to ensure a uniform stress distribution while limiting frictional effects and preventing buckling. For this work, however, rectangular specimens were required to provide a flat surface for the laser-ultrasonics (LUS) measurement [2]. This geometry introduces a non-uniform stress distribution, a known trade-off, but on the other side enhances light collection for the LUS system dramatically. While a comprehensive analysis would require testing both geometries, further optimisation of the specimen design could also mitigate this issue. This study focuses on metal samples, where LUS is employed to gain deeper, in situ insights into material behaviour during deformation [3]. Possible fields of interests are in situ Poisson’s ratio, phase transformation, recrystallisation, hardness penetration depth or defect detection [4,5,6,7,8,9].

LUS testing ideally supplements thermo-mechanical testing, because it enables non-destructive measurement of material properties through laser-induced ultrasonic waves without requiring mechanical contact with the specimen. In the coupled Gleeble-LUS setup considered here, the intended application is non-contact, in situ characterisation during hot compression testing, where ultrasonic information can be correlated with deformation data under variable high-temperature loading conditions. The measured LUS signal contains recurring echoes inside the specimen, which can be used to determine the speed of sound within the material and to infer further material properties. In this context, relevant application targets include in situ elastic-property and defect-related measurements during compression, as highlighted for the integrated Gleeble-laser-ultrasonics platform [10].

During compression testing, the specimen shortens along the compression axis and may expand radially. As a result, the focal points of the LUS excitation and detection beams must be continuously repositioned to remain aligned with the deforming specimen. In the present setup, two motorised axes translate the excitation and detection optics along the compression direction, while a third perpendicular axis adjusts the detection focus according to radial specimen expansion. The engineering problem addressed in this study is therefore the real-time conversion of Gleeble shaft-position information into synchronised LUS-stage motion for maintaining optical alignment during compression [1,11,12,13].

The optical constraints of the detection head make this positioning problem quantitative. For the selected receiver-head geometry, the depth of field (DOF) was derived to be approximately 15 mm, based on geometric optics and the properties of a Lambertian surface. This result was independently confirmed by optical raytracing in Ansys Zemax and by characterisation of the actual device on a temporary mock-up stage setup. Consequently, the stages must act on the detection head if the specimen surface leaves the preset position by more than approximately 7.5 mm during the experiment. In addition, optimal optical coupling requires the opposite sample surfaces between the illumination and return paths, making it desirable to track the centre (on both sides) of the specimen bulge during deformation.

The aim of this study is to develop and evaluate a modular positioning-control system that links the Gleeble 3800 compression (and later on the multi-purpose) assembly with a laser-ultrasonic measurement unit and actively tracks the LUS positions during specimen compression.

The novelty of the proposed approach is the implementation of a dedicated, retrofit-capable acquisition and control chain for LUS focus tracking during compression. Instead of relying on the built-in analogue inputs of the Zaber controllers, which showed a significant noise level for the Gleeble shaft-position signals, the system uses a custom ADC module for digitisation and a Rust-based control architecture for target calculation, stage actuation, process-data access, and user interaction [14,15]. The software also exposes process data through an embedded OPC UA server and provides a browser-based graphical user interface through an integrated HTTP server.

The study addresses the following tasks:

- Define the LUS axis concept required for axial tracking and radial focus compensation during compression;
- Implement a Rust-based, multithreaded control architecture for ADC acquisition, target-position calculation, stage actuation, and data access;
- Integrate OPC UA and HTTP interfaces for process-data exchange, monitoring, and configuration;
- Profile the control loop and reduce latency by reorganising ADC acquisition [16,17].

## 2. Materials and Methods

### 2.1. Experimental Setup

The experimental setup is based on a Gleeble 3800 thermo-mechanical simulator (Dynamic Systems Inc., Poestenkill, NY, USA), which applies compressive force using two hydraulically driven shafts. The system is programmed via a Windows XP workstation and operated by an embedded computer, while the newly developed positioning-control software runs on the separate laser-ultrasonics computer. The positions of the two Gleeble shafts are measured by linear encoders and provided as analogue voltage outputs, one for each shaft. These analogue signals serve as the real-time reference for laser-position tracking during compression.

**Figure 1:**
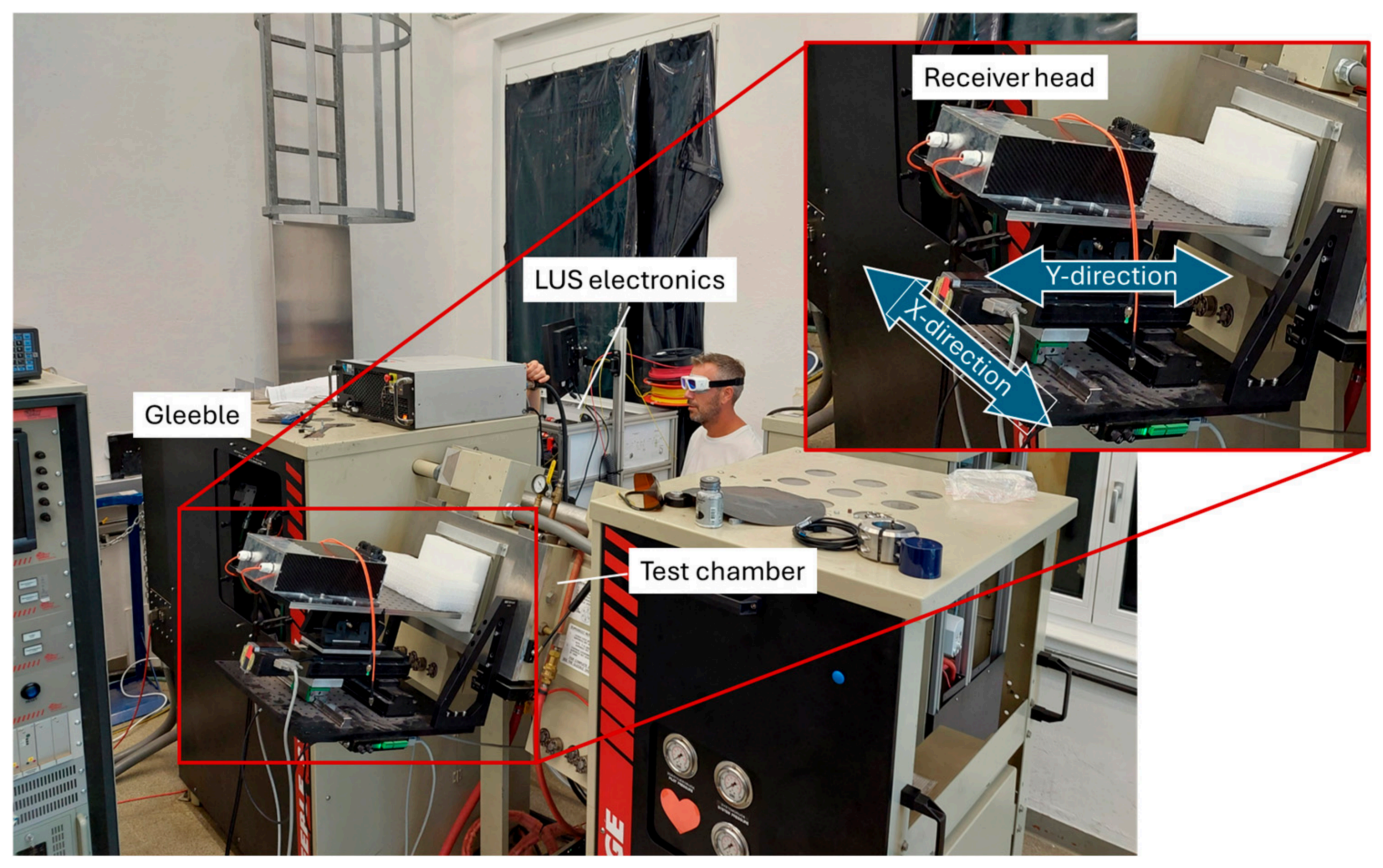
The Gleeble 3800 thermo-mechanical simulator attached with the attached laser-ultrasonic detection unit. The excitation laser and accompanying optics are mounted on opposing sides of chamber on another stage that moves in conjunction with detection unit. Both are positioned outside the vacuum chamber. The measurement is done in a contactless fashion through windows in the chambers access hatches (in figure blocked by white protective foam).

The laser-ultrasonic unit (Figure 1) used in this work (RECENDT GmbH, Linz, Austria) comprises an excitation laser, a detection system (a single-frequency fibre laser, NKT Basik (Hamamatsu Photonics A/S, Hamamatsu, Japan) with three amplifier stages, not visible in the picture), and an industrial computer. To minimise optical losses and surface oxidation, specimens are housed in a vacuum chamber during measurement. The system is employed in a transmissive configuration (detection laser on the opposite sample side of the excitation laser), although a reflective setup is also possible. In this arrangement, the detection laser is connected to the Gleeble via optical fibres and focussed to the specimen during experiments. In this arrangement a measurement specimen was designed to efficiently focus the laser on a small spot and collect the scattered light back into a second fibre. Raytracing software, Ansys Zemax 2014 (ANSYS, Inc., Canonsburg, PA, USA) was used to design and optimise the optical arrangement and to derive the optical parameters, i.e., spot diameter and depth of focus.

**Figure 2:**
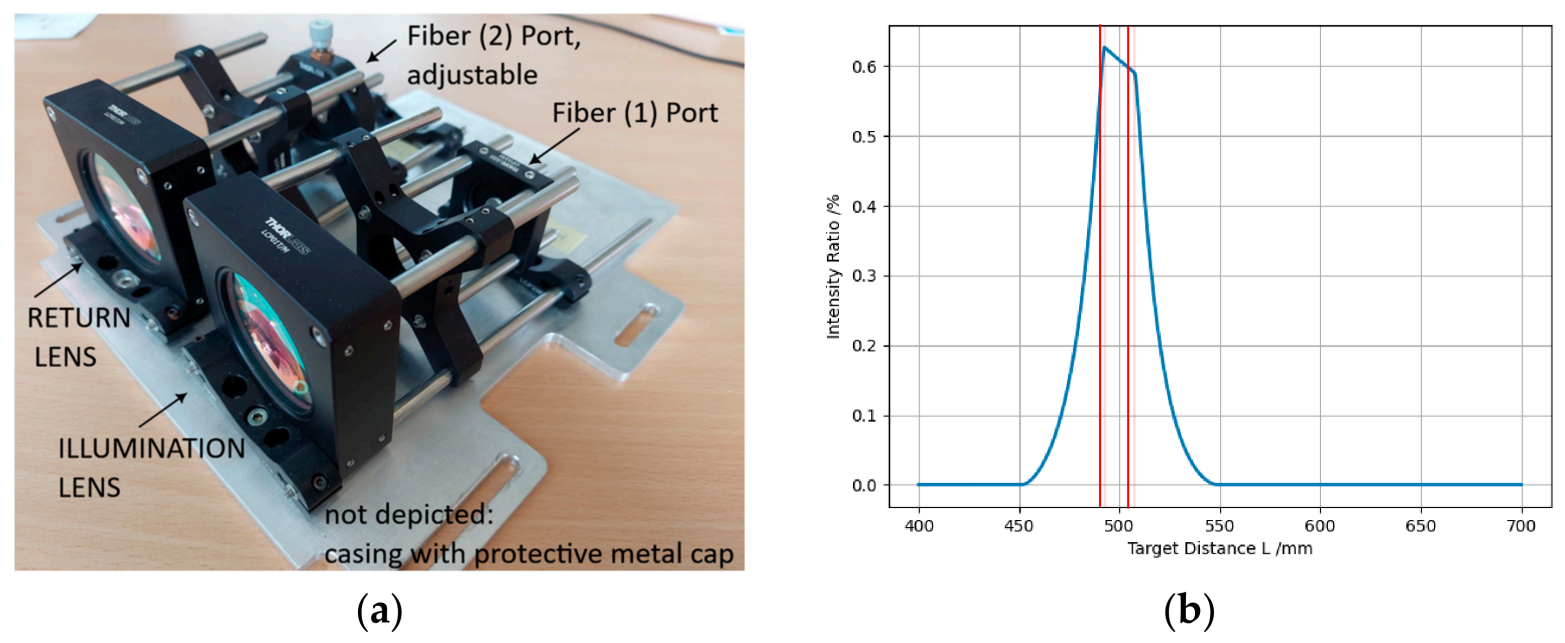
Receiver-head configuration in (**a**) and simplified theoretical model of the detection head, the DOF is highlighted by the red bars in (**b**).

The interferometer receiver head Figure 2a is coupled to the MOPA (master oscillator power amplifier) illumination laser, and to the two-wave-mixing interferometer, by means of multimode optical fibres. The fibre end of the illumination fibre is imaged via a 2-inch lens on the sample surface. Similarly, the specimen reflected light that is then directed into a larger fibre in the return path; specifications shown in Table 1.

From those values, one can derive the depth of field to about 15 mm, see Figure 2b (using geometric optics and the properties of a Lambertian surface [18]), which is appropriate for the given application. The captured energy fraction is generally in the range of 1/100 of the incident energy, dependent on the sample’s surface quality.

### 2.2. Data Acquisition

This result was also reached independently by performing an optical raytracing simulation in Ansys Zemax OpticStudio 2014 R2 [19], and by characterisation of the actual device on a temporary mock-up stage setup.

From this limited depth of field, it is therefore clear that the stages must act upon the detection head were the samples surface to fall outside the preset position by ±7.5 mm at any time during the experiment. In general, efforts are made to keep the distance between the measuring head and the sample as constant as possible, so deviations shall be well within the depth of field, even if the sample was not perfectly centred in the first place.

For most applications, the depth of field is sufficient to cater to plastic deformation, as long as only a single type of sample size is used. Still, due to the constraint that normal incidence shall be maintained, one shall strive to capture the centre of the bulge.

This is also relevant due to the fact that the temperature of the Gleeble takes its readings from the sample’s centre, which may be significantly hotter than the samples edged due to the thermal mass and active cooling of the compression anvil located there.

An interferometer converts phase differences between the probe beam (reflected from the specimen) and a reference beam into an electrical signal. The timing of ultrasonic echoes allows for the measurement of sound velocity and related material properties.

System control is handled by a Windows 10 industrial PC (Intel Core i5-9600K, 16 GB RAM) equipped with a data acquisition (DAQ) card and a microcontroller. Although a custom LabVIEW GUI manages the hardware, its closed-source nature precludes direct integration and hinders automated data handling. This limitation necessitated the development of a standalone software solution to control the linear axes. However, due to the proprietary nature of the main custom LUS software V1.0, measurement files must still be accessed manually after each experiment.

### 2.3. Experimental Procedure and Laser Tracking

Figure 2 illustrates the core principle of laser ultrasonics and the experimental setup. As shown in the schematic (Figure 2), a pulsed excitation laser generates ultrasonic waves on the specimen’s surface [20]. A continuous-wave probe laser reflects from the vibrating surface, and the resulting interference pattern is measured to extract the ultrasonic signal.

During compression tests, the LUS laser focus must be adjusted as the specimen deforms. For this purpose, both the laser and the detector of the LUS unit are mounted on motorised linear axes. Two axes are positioned parallel to the compression direction and move the excitation and detection optics in lockstep, while a third axis is mounted perpendicularly to adjust the detection laser-focus point according to radial specimen expansion, see Table 2. The axes are driven by Zaber control modules, which actuate the stepper motors and provide communication with external software via USB using a purpose-built serial communication protocol.

The Gleeble shaft positions are available as analogue voltage outputs and are therefore suitable as reference signals for stage-position control. Although the Zaber control modules provide built-in ADC inputs, these inputs were found to produce a significant noise level when used for the Gleeble shaft-position signals [21]. A custom analogue-to-digital converter was therefore introduced to provide more reliable digitisation of the two shaft-position signals before they are processed by the control software.

**Figure 3:**
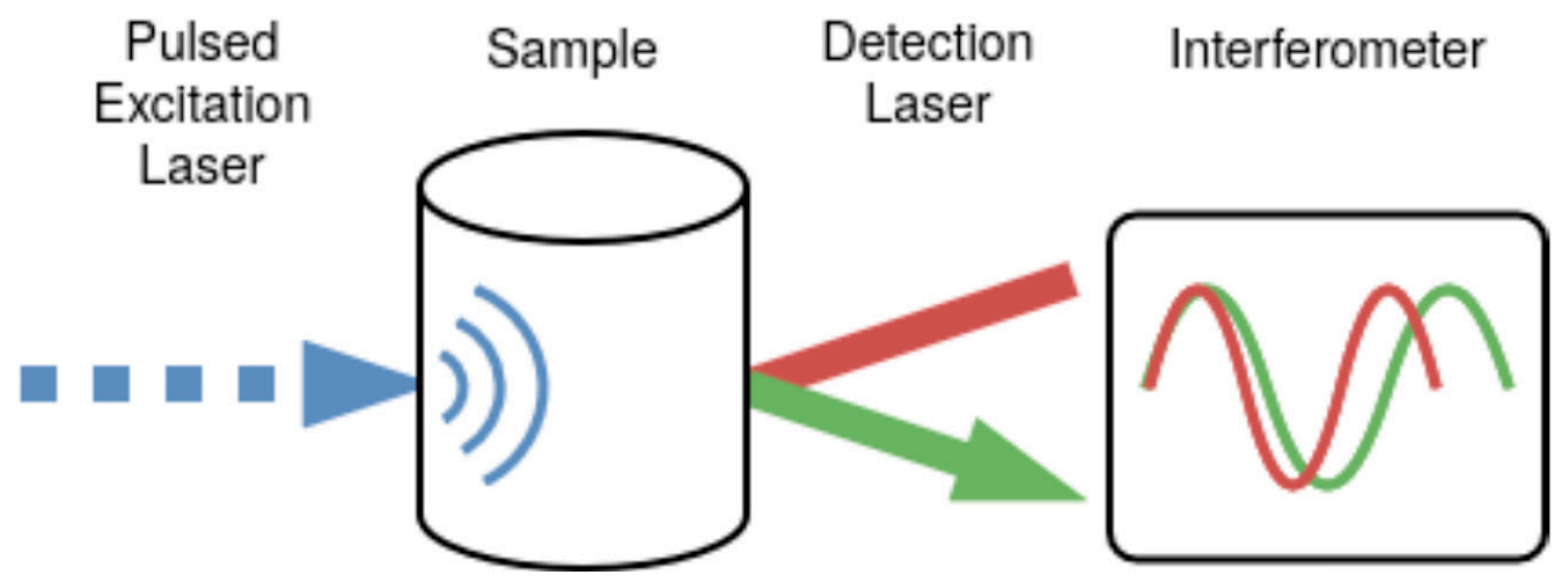
The Pulsed Lasers ablation results, inter alia, in a propagating ultrasonic wavefield through the bulk of the material. The resulting surface deflection on the opposing side is picked up by the vibrometers detection optics. Artistic depiction [15].

The schematic representation of the coupled Gleeble 3800 and laser-ultrasonics positioning setup is shown in Figure 3. The Gleeble applies compression through two hydraulically driven shafts, whose positions are available as analogue voltage outputs from linear encoders. The excitation and detection optics are mounted on motorised linear axes; two axes translate the optics along the compression direction, while a third perpendicular axis adjusts the detection focus according to radial specimen expansion. During compression, the axial axes keep the excitation and detection spots aligned with their predefined specimen locations, while the perpendicular detection axis compensates changes in specimen geometry and the limited depth of field of the detection optics.

**Figure 4:**
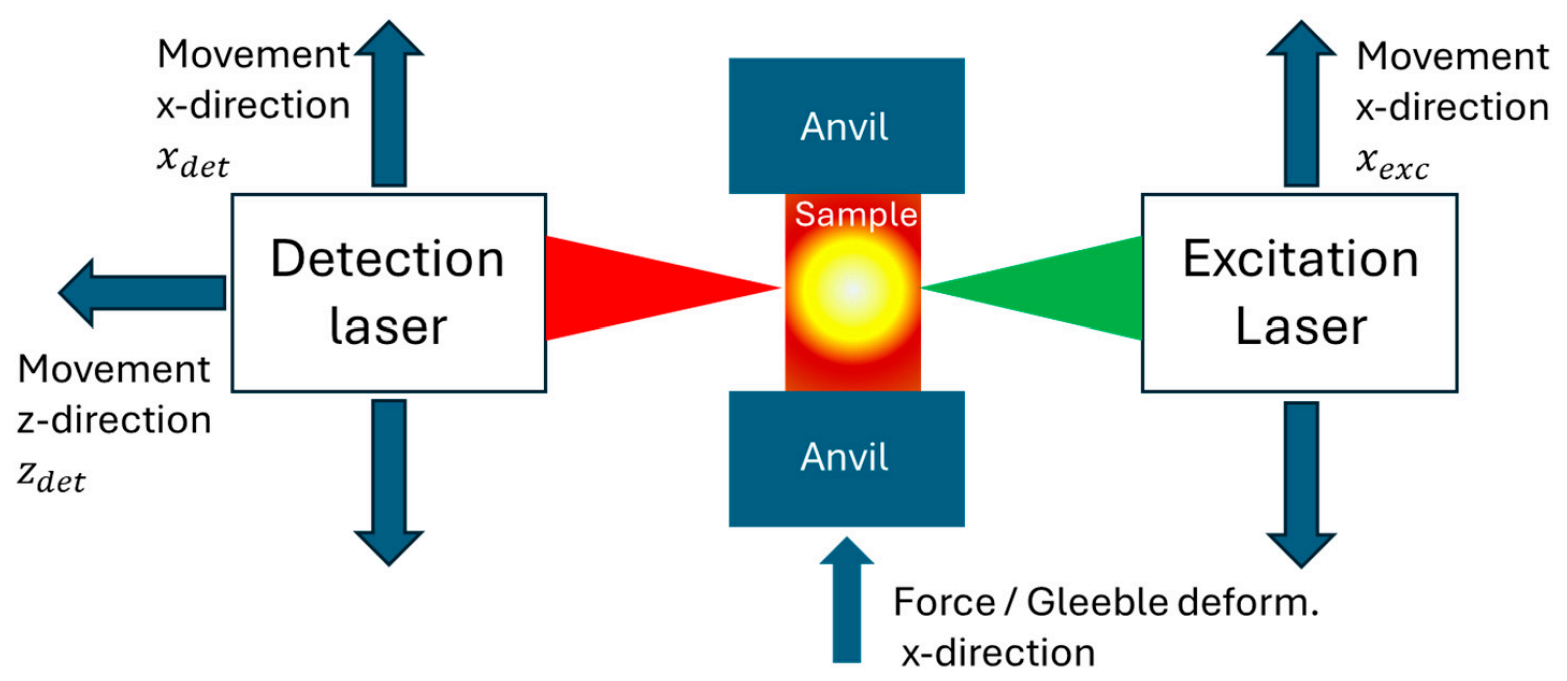
Schematic representation of the coupled Gleeble 3800 and laser-ultrasonics positioning setup.

The need for active positioning is governed by the optical constraints of the detection head. The interferometer receiver head is coupled to the MOPA illumination laser and to the two-wave-mixing interferometer by multimode optical fibres (Figure 4). The illumination fibre end is imaged onto the sample surface using a 2-inch lens, while the sample-reflected light is directed into a larger fibre in the return path. For optimal coupling efficiency, particularly on specularly reflecting surfaces, the sample surface normal should coincide with the half-angle between the illumination and return paths.

For the selected optical design, the nominal working distance is (L = 500 mm), the capturing angle is (α = 11°), the lens focal lengths are (f_1_ = f_2_ = 100 mm), and the fibre core diameters are (d_1_ = 356 µm) for illumination and (d_2_ = 1000 µm) for the return path. From these parameters, the depth of field was derived to be approximately (15 mm). This depth of field is generally sufficient to accommodate plastic deformation when only a single sample size is used, but the stages must correct the detection-head position if the specimen surface moves outside the preset position range of approximately (±7.5 mm).

Therefore, the positioning system must address two coupled effects during compression. First, the excitation and detection spots have to be translated along the compressed specimen axis to keep the investigated volume approximately constant. Second, the detection head must be moved in the radial or focus direction to compensate specimen bulging and maintain the optical coupling condition as closely as possible.

The positioning-control software was implemented in Rust to provide a flexible and high-performance control solution for the coupled Gleeble-LUS setup. Its main task is to acquire the digitised Gleeble shaft-position signals, transform them into laser target positions using configurable mathematical expressions, and command the motorised linear stages accordingly. This design allows the voltage-to-position relationship to be adapted to different specimen geometries, experimental configurations, and calibration procedures without modifying the control software itself.

The target position (p_i_(t)) of each controlled axis (i) is calculated from the current digitised Gleeble voltage signals (V_1_(t)) and (V_2_(t)) using a user-defined expression, see Equation (1).

(1) $$p_{i}\left(t\right)=f_{i}\left(V_{1}\left(t\right),V_{2}\left(t\right)\right)$$

where $i\in \left\{x_{exc},x_{det},z_{det}\right\}$ while $x_{det}$ and $z_{det}$ describe the movement directions of the detection laser while $x_{exc}$ accounts for the axis of the excitation laser. V_1_(t) and V_2_(t) represent the two analogue shaft-position signals provided by the Gleeble, while (f_i_) denotes the configurable conversion expression for the respective axis. The software architecture is multithreaded in order to maintain responsive control and reliable operation while simultaneously handling stage communication, signal acquisition, process-data access, and user interaction.

Process data are made available to external systems through an embedded OPC UA (open platform communications unified architecture) server. OPC UA was selected because the server can be included directly in the positioning-control software without requiring an external message broker, unlike an MQTT-based (message queuing telemetry transport) solution [22]. The OPC UA implementation uses TCP (transmission control protocol) as the underlying protocol to provide high throughput, low latency, and robust performance. In parallel, an integrated HTTP server provides a browser-based graphical user interface for monitoring, manual interaction, and configuration of the positioning system.

The browser-based user interface provides access to the current voltage readings, target positions, actual positions, and configuration parameters of the positioning system. The interface distinguishes between tracking and manual operation modes, allowing the system to either follow the computed target positions or to be moved manually during setup and testing. Configuration fields include the serial device, refresh rate, OPC UA configuration path, target formulas, software limits, maximum speeds, and accelerations for the controlled axes.

## 3. Results

### 3.1. Software Architecture

The runtime of the main control loop was profiled to identify the dominant contributors to the cycle time. In the single-ADC configuration, the probability density distribution of the recorded loop times showed that the control loop was strongly influenced by the ADC-related operations. Flame-graph analysis confirmed that ADC read and write operations accounted for the majority of the execution time.

To reduce this bottleneck, the acquisition was reorganised into a parallel setup. In this configuration, ADC read and write operations still represented the dominant runtime contribution, but the individual operations were shorter, resulting in reduced loop time. The remaining limiting section was associated with a call to usleep inside the libftd2xx library, which makes further optimisation of the main control loop infeasible without changing the low-level communication path.

Rust was selected as the implementation language for this work due to its guarantees of compile-time memory safety and performance comparable to C/C++ [23]. These benefits are achieved through Rust’s ownership and lifetime tracking system, which prevents common errors like use-after-free and dangling pointers without the overhead of a garbage collector [24]. The decision to use Rust was reinforced by an earlier Python V3.13 prototype, which demonstrated significant performance and robustness limitations for the LUS positioning control. Rust ultimately provided a more stable and high-performance solution.

**Figure 5:**
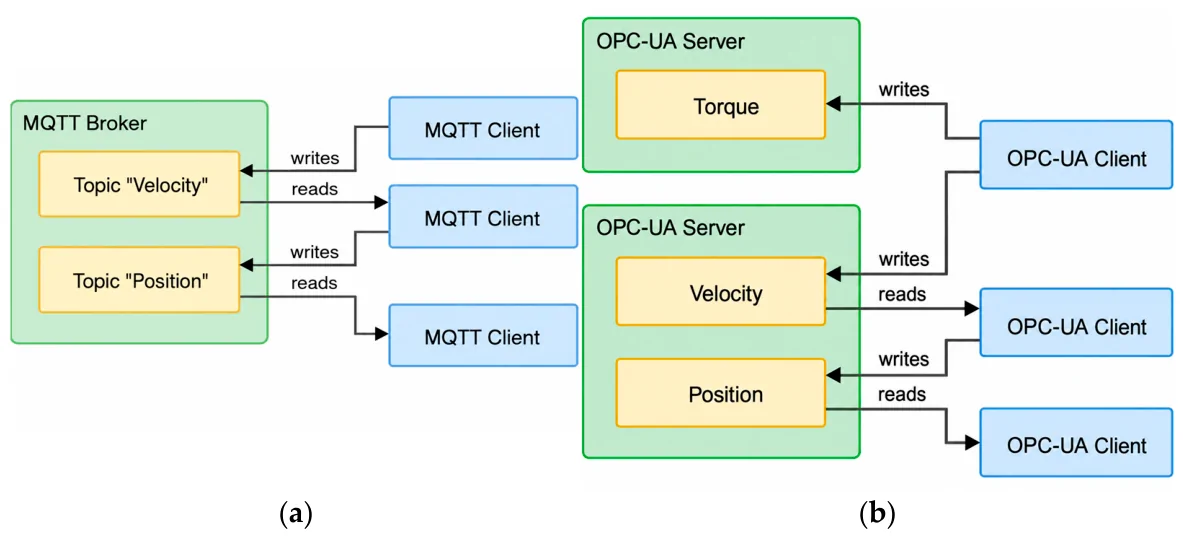
(**a**) MQTT broker–client interaction: the broker manages “Position” and “Velocity” topics; clients read and publish to both. (**b**) Two OPC UA servers expose different process parameters; clients read and write them [15].

For the communication protocol, we evaluated several options. MQTT was deemed unsuitable due to its reliance on an external message broker, which would have introduced an additional dependency on the iba software V3.4.5 packages (Figure 5a) [25].

In contrast, Open Platform Communications Unified Architecture (OPC UA) was chosen because it allows the server to be embedded directly within the positioning-control software, thereby eliminating external dependencies (Figure 5b). Specifically, we implemented OPC UA over TCP to leverage its high throughput, low latency, and robust performance characteristics.

### 3.2. Custom ADC

To drive the LUS axis positioning, a custom ADC module digitises the analogue voltage outputs from the Gleeble shafts. The Zaber controller’s integrated ADC proved too noisy and commercial units were cost-prohibitive. Therefore, a custom solution was developed around an ADS1115 (16-bit, 2–5 V input) employing voltage dividers to scale the Gleeble’s up-to-8 V signals. The ADS1115 communicates via I^2^C; as the LUS PC lacks dedicated I/O headers, an FT232H USB-to-I^2^C adaptor provides the necessary interface. Two identical ADC chains run in parallel to convert the channels of both analogue signals concurrently. To disambiguate the identical FT232H devices at start-up, one module incorporates an extra 3 V ID line. These components are illustrated in Figure 5a,b.

The positioning-control software is structured around three concurrent threads: (1) a control unit executing the real-time loop, (2) an OPC UA server for external data access, and (3) an HTTP server that provides a browser-based graphical user interface (GUI). The control unit operates as a state machine (stopped, initialisation, running, clean-up) and supports both tracking and manual modes. In tracking mode, it reads voltages via the ADC, transforms them into target positions using user-defined formulae and sends commands to the Zaber controller. System status is periodically polled and shared. To ensure low-latency concurrency thread-safe read/write locks and channels are employed. Configuration is managed through a TOML (Tom’s Obvious Minimal Language) file, which can be edited via the web GUI when the system is stopped.

### 3.3. Optimisation

**Figure 6:**
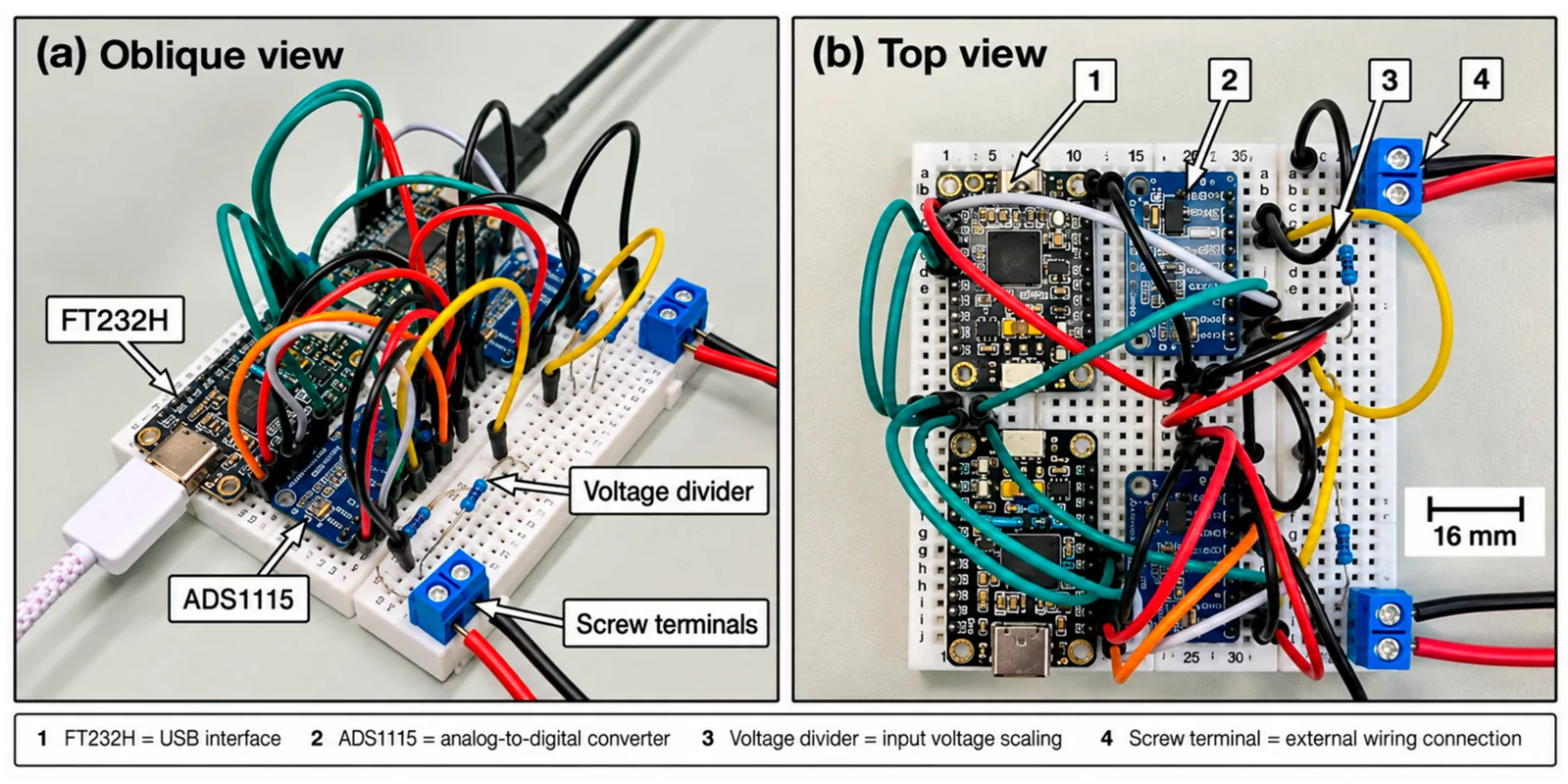
Showing the custom-built ADC module and its components. (**a**) Side view and (**b**) top view.

Profiling of the main control loop using flame graphs revealed that ADC I/O dominated the runtime (≈90%), with delays attributable to vendor driver calls (usleep in libftd2xx). Rather than rewriting the drivers, the performance through parallelisation has been improved. By implementing two ADS1115 chains on separate USB ports, we could read both analogue channels concurrently, with the operation executed by a parallel iterator. Dedicating an ADC to each channel also enabled continuous-conversion mode, as opposed to one-shot mode, further reducing overhead. Consequently, the mean loop time decreased from approximately 130 ms to 27 ms (Figure 6a,b). The primary remaining bottlenecks are these driver-induced sleeps, which limit further performance gains.

**Figure 7:**
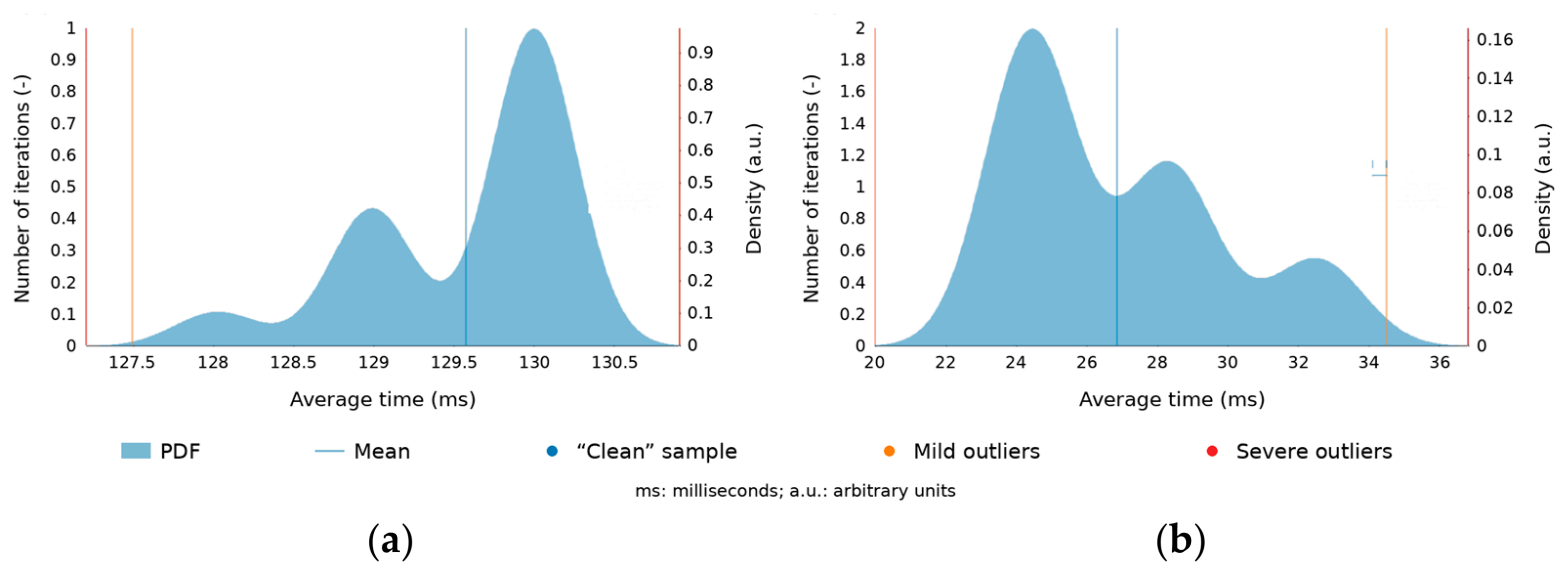
Probability density distributions of the main control-loop execution time. (**a**) Runtime distribution for the single-ADC configuration. (**b**) Runtime distribution for the parallel acquisition configuration.

To ensure correctness and robustness, we implemented both unit and integration testing. While unit tests cover critical control logic, integration tests validate the browser-based GUI using Playwright. A purpose-built simulator emulates the Zaber controller and Gleeble analogue signals, which enables hardware-independent, automated regression testing on any machine following each code change. Profiling showed that ADC read and write operations dominate the execution time in both configurations, while the parallel setup reduces the duration of these operations and therefore lowers the overall loop time, Figure 7a,b.

The implemented software was structured to separate signal acquisition, target-position computation, stage actuation, process-data exposure, and user interaction. This modular structure supports future extensions and adaptation to changing experimental requirements. The browser-based interface provides controls for starting and stopping the positioning system, displaying voltage readings, and showing target and actual positions of the controlled axes. In addition, the configuration interface exposes target formulas, axis limits, maximum speeds, accelerations, and communication settings, allowing the system to be adapted to different experiment configurations without recompilation.

## 4. Discussion

The developed positioning system addresses the central experimental challenge of maintaining laser alignment on a deforming specimen during compression testing. The axial motion of the excitation and detection optics compensates specimen shortening and helps to keep the interrogated volume approximately constant. The perpendicular detection-axis motion compensates radial expansion and focus changes, which can be necessary because the detection optics provide only a limited depth of field of approximately (15 mm). By using the Gleeble shaft-position voltages as reference signals, the system links the mechanical deformation state of the specimen directly to the optical alignment of the LUS measurement.

The custom ADC-based acquisition chain is a key part of this link. The built-in ADC channels of the Zaber control modules were initially considered for reading the Gleeble shaft-position signals, but the observed noise level made them unsuitable for reliable position feedback. The developed software therefore digitises the shaft-position signals through a custom ADC and transforms them into target positions using configurable mathematical expressions. This configurability is important because the relation between shaft position, specimen geometry, and required laser position depends on the experimental configuration.

The communication architecture was designed to support integration into a broader experimental and data-acquisition environment. OPC UA was chosen because it combines communication, information modelling, and security in a single industrial standard. In contrast to an MQTT-based approach, the OPC UA server can be embedded directly into the positioning-control software and does not require a separate external message broker. This reduces the number of required infrastructure components and simplifies operation in the experimental setup. The integrated HTTP server complements this by providing a browser-based interface for configuration and monitoring.

The performance analysis shows that the dominant limitation of the control loop lies in the ADC communication path rather than in the control computation itself. Flame-graph analysis indicated that ADC read and write operations required the majority of the execution time. The parallel acquisition setup reduced the duration of these operations and therefore reduced loop times, but the remaining bottleneck was traced to a usleep call inside the libftd2xx library. This indicates that further improvements would likely require changes to the low-level acquisition hardware or driver stack rather than only optimisations of the high-level control logic.

Several limitations remain. First, the finite depth of field of the detection optics defines a limited tolerance window around the preset focus position. Although the approximately 15 mm depth of field is generally sufficient for many applications when only one sample size is used, active correction is required if the specimen surface moves outside the approximately ±7.5 mm range during the experiment. Second, coupling efficiency is maximised when the specimen’s surface normal corresponds to the angle bisector between the illumination and detection path. Third, the excitation laser can cause accumulating damage at its incident spot on the sample over a few hundred measurements, which may influence long experimental sequences or repeated measurements at the same location. The ultrasound generation is an ablative process, leading to accumulating damage. In this case, when using a q-switched excitation laser of up to 100 mJ (Quantel Merion C, at a maximum of 400 Hz pulse repetition rate) on a spot of 4 mm diameter (fluence up to 800 mJ/cm^2^), the damage will become clearly noticeable after seconds, corresponding to a few hundred shots and several micrometre deep cratering. The ablation of material is in the sub-µm range per shot. It is considered good practice to limit the number of pulses to a few hundred where applicable as to not introduce extra complexity into the measurement. The different contributions to the error-budget are shown in Table 3. In the literature comparable studies can be found as in [26].

Fourth, the current loop-time limitation is partly associated with the low-level ADC communication path and the libftd2xx driver behaviour, as indicated by the profiling results. Future work should therefore focus on quantitative tracking-error measurements during real compression experiments, systematic evaluation of LUS signal quality with and without active tracking, and further reduction in acquisition latency if higher tracking rates are required.

A full quantitative separation of Gleeble machine error, stage-positioning error, ADC uncertainty, and dynamic tracking error was not performed in this study and requires dedicated calibration and in situ validation experiments. This limitation has been added as future work.

## 5. Conclusions

The developed system achieved active laser-focus tracking for coupled Gleeble 3800 compression and laser-ultrasonic measurements by using the Gleeble analogue shaft-position outputs as real-time reference signals. During compression, two motorised stages compensate specimen deformation along the compression axis, while a perpendicular stage compensates radial expansion and focus changes caused by the limited depth of field of the detection optics.

The acquisition chain was implemented with a custom ADC while the built-in ADC channels of the Zaber controllers showed excessive noise and were therefore unsuitable for reliable position feedback. The digitised shaft-position signals are converted into target positions by user-configurable mathematical expressions and then sent to the motorised stages.

The main quantitative result is the reduction in the control-loop latency from about 130 ms to about 27 ms using the Rust-based control system with custom analogue-to-digital acquisition. Profiling showed that ADC read/write operations dominate the loop runtime, that parallel acquisition reduces the duration of these operations, and that the remaining bottleneck is associated with usleep calls in the libftd2xx driver path.

The implementation also achieved automated validation and flexible operation. Unit tests cover critical control functions, integration tests validate the browser-based GUI, and a simulator emulates the Zaber controller and Gleeble analogue signals to allow hardware-independent regression testing. The system exposes process data through OPC UA and provides configuration and monitoring through an integrated HTTP-based graphical interface.

The remaining limitations are mainly experimental and low-level communication constraints. The detection optics have a depth of field of approximately 15 mm, so active correction is required when the specimen surface moves outside the approximately ±7.5 mm tolerance range. Repeated measurements also cause accumulating laser damage after a few hundred measurements; the used Quantel/Lumibird Merion C laser which operates at 400 Hz with 100 mJ pulses. Future work should quantify tracking errors during real compression tests, compare LUS signal quality with and without active tracking, and further reduce acquisition latency if higher tracking rates are required.

## Figures and Tables

**Table 1:** The chosen parameters of the interferometer receiver head.

Nominal working distance	L	500 mm
Capturing aperture	A	$A=\pi r^{2}=\pi \times {\left(25.4\;mm\right)}^{2}$
Capturing angle	alpha	11°
Lenses focal length	f1, f2	100 mm
Fibre core diameters	d1, d2	356 µm (Illumination) 1000 µm (Return)
Fibre numerical aperture	NA1, NA2	0.22, 0.39

**Table 2:** Axis nomenclature used for the laser-ultrasonic positioning system with Zaber modules. Two axes are arranged parallel to the compression direction to move the laser and detector in lockstep, while the third axis is mounted perpendicularly to adjust the detection laser-focus point according to radial specimen expansion.

Component	Model Name	Manufacturer
Linear Axis	LRT0100BL-CT3	Zaber (Zaber Technologies Inc., Vancouver, BC, Canada)
Axis Control Module (large)	X-MCB2	Zaber
Axis Control Module (small)	X-MCB1	Zaber

**Table 3:** Comparison of the present work with related positioning/error-compensation approaches.

Error-Budget Contribution	System Origin	Effect on Laser Tracking	Status in This Work
Shaft-position uncertainty	Linear encoders and analogue voltage outputs	Error in deformation-state reference	Identified as part of the reference-signal chain
Analogue voltage noise	Gleeble voltage output and cabling	Fluctuation of computed target positions	Reduced by replacing noisy Zaber inputs with a custom ADC
ADC digitisation uncertainty	Custom ADC acquisition chain	Noise in $V_{1}\left(t\right)$ and $V_{2}\left(t\right)$	Acquisition chain implemented; numerical budget requires calibration
Voltage-to-position uncertainty	User-defined functions $f_{i}=\left(V_{1},V_{2}\right)$	Model/calibration error in target position	Configurable formulas allow adaptation to specimen geometry and calibration
Control-loop latency	ADC read/write operations and driver delays	Dynamic following error during deformation	Mean loop time reduced from 130 ms to 27 ms using parallel acquisition
Stage-positioning uncertainty	Zaber linear stages and controllers	Difference between applied and actual optical position	Identified; full numerical quantification requires stage calibration
Optical focus tolerance	Limited depth of field of the receiver optics	Reduced LUS signal quality if focus deviates too far	DOF approximately 15 mm; correction needed beyond approximately ±7.5 mm
Specimen shape uncertainty	Shortening and radial expansion during compression	Difference between assumed and actual focus location	Specimen shapes can be investigated after testing

## Data Availability

The datasets presented in this article are not readily available because of the underlying thesis which is not published yet. Requests to access the datasets should be directed to the corresponding author.

## References

[1] Prabitz K.M., Walzl A., Stockinger M. (2026). Mathematically Compensating for the Barrelling Effect Occurring During Compression Testing of Additive-Manufactured A20X Samples and Describing Friction with Validated Finite Element Models. Appl. Mech..

[2] Monchalin J.-P. (2020). Laser-Ultrasonics: Principles and Industrial Applications. http://www.ndt.net/?id=25250.

[3] Saba N., Jawaid M., Sultan M.T.H. (2018). An overview of mechanical and physical testing of composite materials. Mechanical and Physical Testing of Biocomposites, Fibre-Reinforced Composites and Hybrid Composites.

[4] Sellars C.M., Whiteman J.A. (1979). Recrystallization and grain growth in hot rolling. Met. Sci..

[5] Hartl K., Sorger M., Stockinger M. (2023). The Key Role of Laser Ultrasonics in the Context of Sustainable Production in an I 4.0 Value Chain. Appl. Sci..

[6] Militzer M., Hoyt J.J., Provatas N., Rottler J., Sinclair C.W., Zurob H.S. (2014). Multiscale modeling of phase transformations in steels. JOM.

[7] Militzer M., Maalekian M., Moreau A. (2012). Laser-ultrasonic austenite grain size measurements in low-carbon steels. Mater. Sci. Forum.

[8] Watzl G., Kerschbaummayr C., Schagerl M., Mitter T., Sonderegger B., Grünsteidl C. (2022). In situ laser-ultrasonic monitoring of Poisson’s ratio and bulk sound velocities of steel plates during thermal processes. Acta Mater..

[9] Haderer W., Scherleitner E., Gseller J., Heise B., Mitter T., Ryzy M., Reitinger B., Hettich M. (2023). Spatial imaging of stratified heterogeneous microstructures: Determination of the hardness penetration depth in thermally treated steel parts by laser ultrasound. NDT E Int..

[10] Hutchins D.A. (1988). Ultrasonic Generation by Pulsed Lasers. Phys. Acoust..

[11] Vorobyev A., Bjurhager I., van Dijk N.P., Gamstedt E.K. (2016). Effects of barrelling during axial compressive tests of cubic samples with isotropic, transversely isotropic and orthotropic elastic properties. Compos. Sci. Technol..

[12] Khoddam S., Mansourinejad M., Mirzakhani B., Hodgson P.D., Taylor A.S. (2022). A comprehensive experimental and numerical study of foldover onset and growth during the barrelling compression test. Adv. Mech. Eng..

[13] Khoddam S., Hodgson P.D. (2021). Conversion of compression test data into flow curve, accounting for barrelling. Comput. Methods Mater. Sci..

[14] Zaber Technologies, Inc. ASCII Protocol Manual. https://www.zaber.com/protocol-manual?protocol=ASCII.

[15] Schwarz M. (2026). Laser-Ultrasonics Positioning Control for Compression Testing. Master Thesis.

[16] Zhang Y., Zhang Y., Portokalidis G., Xu J. (2022). Towards Understanding the Runtime Performance of Rust. ACM International Conference Proceeding Series.

[17] Santoso O.K.A., Kwee C., Chua W., Nabiilah G.Z., Rojali (2023). Rust’s Memory Safety Model: An Evaluation of Its Effectiveness in Preventing Common Vulnerabilities. Procedia Comput. Sci..

[18] Hecht E. (2017). Optics.

[19] Synopsys, Inc., ANSYS, Inc. Ansys Zemax OpticStudio. https://ansys.synopsys.com/de-de/products/optics/ansys-zemax-opticstudio.

[20] Grünsteidl C., Veres I., Berer T., Kreuzer S., Rothemund R., Hettich M., Scherleitner E., Ryzy M. (2020). Measurement of the attenuation of elastic waves at GHz frequencies using resonant thickness modes. Appl. Phys. Lett..

[21] Zaber Technologies, Inc. X-MCB2 Series User’s Manual. https://www.zaber.com/manuals/X-MCB2.

[22] Hanenberg S., Kleinschmager S., Robbes R., Tanter É., Stefik A. (2013). An empirical study on the impact of static typing on software maintainability. Empir. Softw. Eng..

[23] Bhattacharya P., Neamtiu I. (2011). Assessing Programming Language Impact on Development and Maintenance: A Study on C and C++. ICSE ‘11: Proceedings of the 33rd International Conference on Software Engineering.

[24] Edelson D.R. (1992). Smart Pointers: They’re Smart, but They’re Not Pointers.

[25] Soni D., Makwana A. (2017). A Survey on MQTT: A Protocol of Internet of Things(IOT). International Conference on Telecommunication, Power Analysis and Computing Techniques (ICTPACT-2017).

[26] Díaz-Tena E., Ugalde U., López de Lacalle L.N., de la Iglesia A., Calleja A., Campa F.J. (2013). Propagation of assembly errors in multitasking machines by the homogenous matrix method. Int. J. Adv. Manuf. Technol..

